# A novel AI-coupled flow chamber method quantifying erythrocyte osmotic fragility

**DOI:** 10.1038/s41598-026-38322-z

**Published:** 2026-02-17

**Authors:** Ipek Seda Fırat, Özgür Alaçayır, Till Creutz, Gerhard Michael Artmann, Samar Damiati, Ayşegül Temiz Artmann

**Affiliations:** 1 Center of Competence for Bioengineering, University of Applied Sciences Aachen, Medical and Biological Laboratory, Jülich, Germany; 2 HiTec Zang GmbH, TPH Technology Park Herzogenrath, Aachen, Germany; 3 Department of Chemistry, College of Sciences, University of Sharjah, Sharjah, United Arab Emirates

**Keywords:** Anemia, Sepsis, Osmotic fragility, Erythrocytes, Blood banking, Artificial intelligence, Biochemistry, Biological techniques, Biophysics, Biotechnology

## Abstract

Osmotic fragility (OF) is widely used to evaluate red blood cell (RBC) membrane stability, water transport dynamics and hemoglobinopathies, traditionally via spectrophotometric, visual, or flow cytometric techniques. Here, we present a novel flow chamber-based platform integrated with a proprietary imaging software providing AI-driven RBC detection for automated and reproducible OF analysis (BioExP). To assess agreement, we compared MCF₅₀ values from four healthy donors using classical and flow chamber methods. Protocol optimization included determining the “satiation time” (optimal incubation required to induce maximal hemolysis without overexposure) and hemolysis kinetics. Biological sensitivity was tested using two modulators: HgCl₂ (40 µM) to inhibit aquaporin (AQP) channels and lipopolysaccharide (LPS, 1000 µg/mL) to increase membrane fragility. Both treatments caused significant shifts in MCF₅₀ when compared to control: AQP inhibition decreased MCF₅₀ to 0.37 ± 0.01% NaCl (flow chamber) and 0.40 ± 0.01% NaCl (classical), while LPS increased MCF₅₀ to 0.44 ± 0.01% NaCl and 0.47 ± 0.004% NaCl, respectively (*p* < 0.001 for all). The BioExP replicated classical OF measurements, captured donor-specific variability, and detected changes. Importantly, it demonstrated that LPS alone, in plasma-free conditions, can compromise RBC membrane integrity. The platform requires minimal sample volume and enables real-time imaging and multi-condition testing.

**Supplementary Information:**

The online version contains supplementary material available at 10.1038/s41598-026-38322-z.

## Introduction

The osmotic fragility (OF) test, first introduced in 1946, remains a widely used method to assess the integrity and mechanical resilience of red blood cells (RBCs) under hypotonic stress^1,2^. In this assay, RBCs are subjected to a series of decreasing sodium chloride (NaCl) concentrations, and the extent of hemolysis is quantified, typically by spectrophotometric analysis of free hemoglobin in the supernatant. The results are often expressed as the mean corpuscular fragility (MCF_50_), which corresponds to the NaCl % at which 50% hemolysis occurs. In addition to standard spectrophotometric approaches, alternative techniques such as naked-eye assessments, refractometry, turbidimetry and flow cytometry have been employed in both clinical and research settings^3–6^.

Originally developed for hematological diagnostics of hereditary spherocytosis, a condition marked by spherical erythrocytes with reduced deformability that lyse more readily in hypotonic environments, it is now also applied in detecting and studying thalassemia, anaemias, and other hemoglobinopathies where membrane integrity and cellular water handling are altered.^4,6–14^

Beyond its diagnostic utility in humans, the OF test has broad applicability in veterinary hematology, toxicology, and transfusion medicine^15–22^. It has been used to assess oxidative stress, the hemolytic potential of novel pharmaceuticals and environmental agents, and storage-related changes in RBC quality^16,17,21,23–25^. In basic research, time-limited OF is a valuable method for exploring cellular water dynamics and aquaporin (AQP) functionality^26–30^. AQPs are integral membrane proteins that enable water movements across cell membranes, thereby contributing critically to the regulation of cell volume homeostasis^31–34^. Among the most commonly used inhibitor in AQP studies is mercury chloride (HgCl_2_), which at sub-toxic concentrations can reduce time-limited RBC fragility by limiting water permeability^26,27,32,35–37^. In contrast to reducing RBC fragility through AQP modulation , several factors have been shown to increase fragility, including exposure to cold or hyperthermic temperatures, increase in pH, certain anticoagulants (e.g., EDTA, citrate), alterations in membrane composition, and oxidative membrane damage^7,8,12,38–40^. Another factor proposed to increase osmotic fragility is lipopolysaccharide (LPS), an endotoxin believed to destabilize erythrocyte membranes via oxidative and inflammatory mechanisms^41–44^. However, the extent of this effect is likely dependent on the specific LPS type and incubation duration. Notably, the current literature on the direct impact of LPS on RBC osmotic fragility is limited. All in all, the OF test is highly sensitive to a wide range of extrinsic factors, including experimental conditions, sample handling, storage time, centrifugation speed, pH, and temperature, as well as intrinsic factors like species, age, and sex, all of which can significantly affect the outcomes.^5,8,18,24,39,40,45–55^. These sensitivities underscore the importance of testing for OF-modulating factors under controlled conditions, and highlight the need for advanced platforms that enable more comprehensive and high-throughput OF analysis.

In recent years, only a few lab-on-a-chip and microfluidic platforms have been developed for assessing osmotic fragility, typically relying on camera-based image acquisition and custom software such as MATLAB for image processing^10,56^. While these systems offer miniaturization and automation, they often remain limited in scalability, accessibility, or real-time analysis.

In this study, we present a novel, AI based, microfluidic flow chamber device for time-limited OF assessment, called BioExP, integrating a programmable pump system, temperature control, real-time imaging, and proprietary AI-based analysis software. To explore its potential, we conducted comparative analysis using blood from healthy donors, with the classical OF method. To demonstrate biological relevance and sensitivity, we additionally employed two modulators of RBC osmotic resistance: LPS to increase osmotic fragility, and HgCl_2_ to reduce it via AQP inhibition. This allowed us to demonstrate the system’s ability and sensitivity to detect shifts in osmotic fragility under physiologically relevant conditions. Our findings showed that AQP inhibition significantly lowered fragility where as LPS incubation in the absence of plasma increased fragility under tested concentrations and incubation protocols^57^. More importantly, these results support the feasibility and utility of the BioExP-OF method as a powerful tool for biological, preclinical and clinical studies, particularly in the field of hemolytic anemia,as well as for basic research applications requiring flexibility, speed and multi-condition testing.

## Results and discussion

### The flow chamber platform, monolayer technique and image analysis

**Fig. 1:**
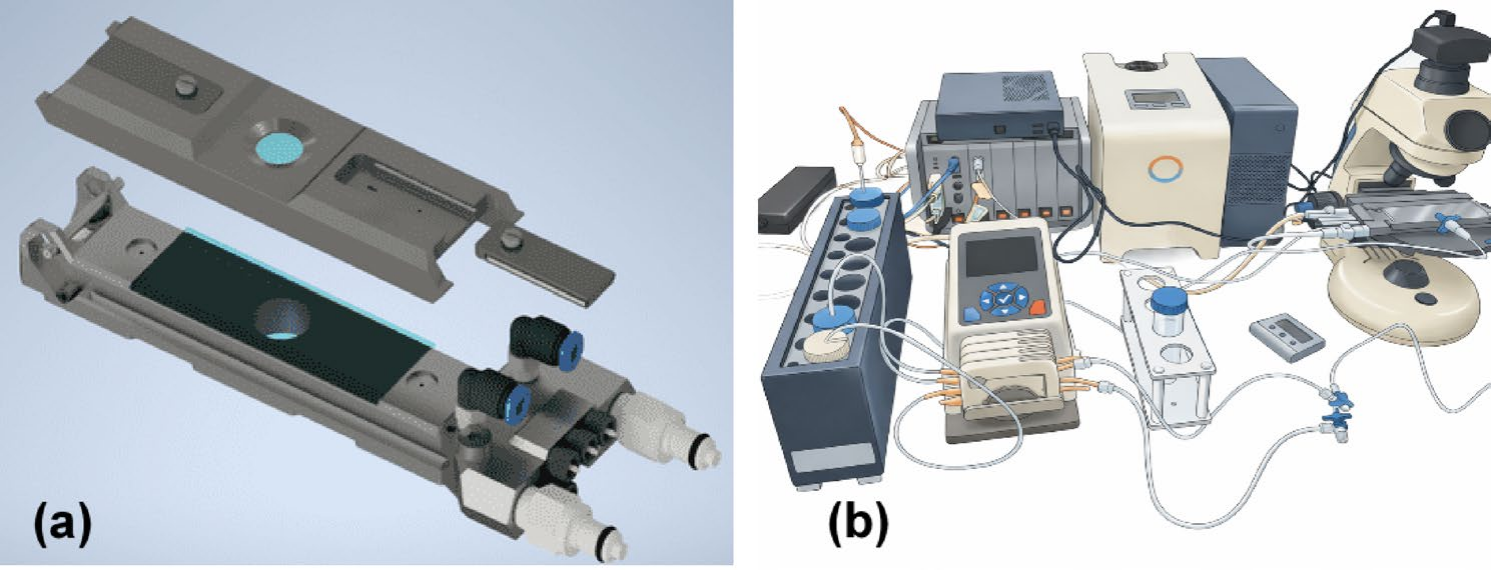
**(a)** Schematic design of the flow chamber system used for osmotic fragility assessment. **(b)** Schematic illustration of the BioExP experimental setup used for osmotic fragility measurements. The system integrates temperature control, perfusion, flow-chamber, microscope for assessment of OF. The illustration is a simplified representation of the experimental arrangement and is not drawn to scale.

Figure 1A depicts the flow chamber design with the circular window for real-time visualization covered with thin, hardened glass (0.2 mm), pneumatic fast coupling for inlet/outlet valves (black), valved fast-coupling for tempered water (white), three inlets for fast fluid exchange (dark grey). This set-up, connected to the programmable pump, allowed controlled exposure of hypotonic solutions to the RBC monolayer. The flow chamber is secured on the table of the microscope for the entire process of the measurement. Figure 1B, is showing the flow chamber setup.

**Fig. 2:**
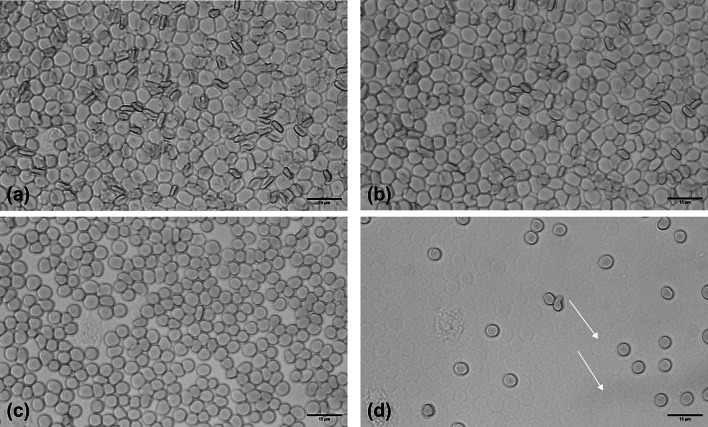
Microscopic images of RBC monolayers in the flow chamber showing the progression of RBC hemolysis at decreasing NaCl concentrations, captured in the same field of view over time. Arrows depict ghost cells. **(a)** RBC monolayer at baseline, NaCl concentration of 0.9% (w/v), **(b)** After 3 min of incubation with 0.7% NaCl ( w/v), **(c)** After 3 min of incubation with 0.5% NaCl (w/v), **(d)** After 3 min of incubation with 0.4% NaCl (w/v).

Figure 2 illustrates the progressive hemolysis of RBCs under increasing hypotonic stress in the flow chamber setup. At baseline (0.9% NaCl, w/v), a uniform RBC monolayer is visible, serving as the reference for subsequent measurements (Fig. 2a).

Following an incubation step for 3 min in 0.7% NaCl (b), it is evident that the cells begin to adopt a more spherocytic shape, with the central indentation (visible as a darker region, Fig. 2a) diminishing due to cellular swelling. At 0.5% NaCl the number of intact RBCs is noticeably reduced, indicating ongoing hemolysis. By 0.4% NaCl, substantial cell loss is evident, and residual ghost cells as remnants of lysed RBCs remain attached to the slide surface, highlighted by white arrows (Fig. 2d). These images visually confirm the concentration-dependent nature of osmotic fragility, and are used to assessed OF with the help of the AI.

### Satiation time analysis

**Fig. 3:**
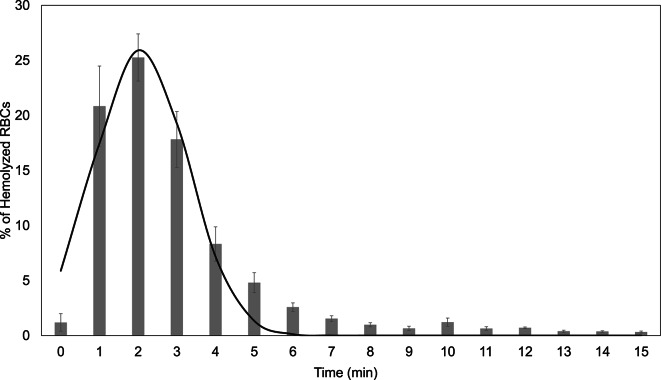
Percentage of hemolyzed RBCs over incubation time (min) (average % of hemolyzed RBCs ± SEM). The line represents a Gaussian fit of the data, with a goodness of fit of 93.91%, n = 8.

In the context of this paper, ‘satiation time’ refers to the incubation period at which hemolysis reaches a steady state. This analysis was employed to determine the optimal incubation duration required to capture the maximum hemolysis rate in the flow chamber system. This step was critical to avoid both underexposure and oversaturation of RBCs to osmotic stress, which could artificially increase or decrease MCF_50_. Figure 3 illustrates the percentage of RBCs lost from the monolayer following exposure to 0.4% NaCl (w/v) over a 15-min period.

The peak hemolysis rate was observed at approximately 2 min, corresponding to a maximum lysis of 25.95%, as determined by the Gaussian fit. The lag phase began immediately after exposure (~ 0 min), aligning with literature that suggests hemolysis begins within seconds to sub-seconds of exposure to osmotic stress^58,59^. On average, ~ 77% of all RBCs that would lyse during the full 15-min assay did so within the first 3 min. The active hemolysis window, defined as the period between the onset and decline of rapid hemolytic activity, spanned from 0.65 to 3.49 min, highlighting the dynamic osmotic response during this interval.

In the presented dataset the resulting hemolysis kinetics follow a left-skewed Gaussian profile, with the 3-min time point lying on the descending phase of the curve. Moreover, during assay development, preliminary tests with different incubation times (data not shown) were performed, which also indicated that a 3-min incubation provided sufficient hemolysis for reliable curve fitting while keeping the overall assay duration short. This timing captures a strong hemolytic response without reaching full saturation and thereby helps to avoid overestimation of MCF₅₀. Together, these observations indicate that most osmotically induced hemolysis occurs rapidly within the first few minutes of exposure.

### Baseline MCF_50_ comparison between classical and flow chamber methods

Descriptive statistical data for the MCF_50_ values obtained using the classical and the flow chamber methods are summarized in Table 1. Both methods showed comparable mean MCF_50_ values (0.41% NaCl) and exhibited similar variability, as indicated by equivalent SEM and SD values. The CV % were also closely aligned (CV = 5.19% vs 5.21%). The IQR was slightly wider for the flow chamber method (0.04) compared to the classical method (0.03), suggesting marginally greater variability in the central distribution of the measurements. The 95% confidence intervals for both methods were narrow (± 0.01), indicating a high degree of precision in the estimation of the mean MCF_50_ values.

**Table 1:** Descriptive statistics of MCF₅₀ values obtained using the classical and flow chamber osmotic fragility methods. Data are presented as mean and median with associated measures of variability. Standard deviation (SD) reflects variability across measurements, while the standard error of the mean (SEM) represents the uncertainty of the estimated mean. Minimum and maximum values denote the observed range, with the range defined as maximum minus minimum. The interquartile range (IQR) is defined as the difference between the 75th and 25th percentiles, and the 95% confidence interval (CI) was calculated for the mean. The coefficient of variation (CV %) was calculated as SD divided by the mean. (n = 24 paired measurements (4 donors × 6 repeated measurements per donor); repeated-measures design.

Statistic	Classical OF Method	Flow Chamber OF Method
Mean MCF_50_	0.41	0.41
Standard Deviation (SD)	0.02	0.02
Standard Error of the Mean (SEM)	0.004	0.004
Minimum	0.37	0.38
Maximum	0.44	0.45
Range	0.07	0.07
Median	0.42	0.40
Variance	0.0004	0.0005
Interquartile Range (IQR)	0.03	0.04
95% Confidence Interval	± 0.01	± 0.01
Coefficient of Variation (CV%)	5.19	5.21

**Fig. 4:**
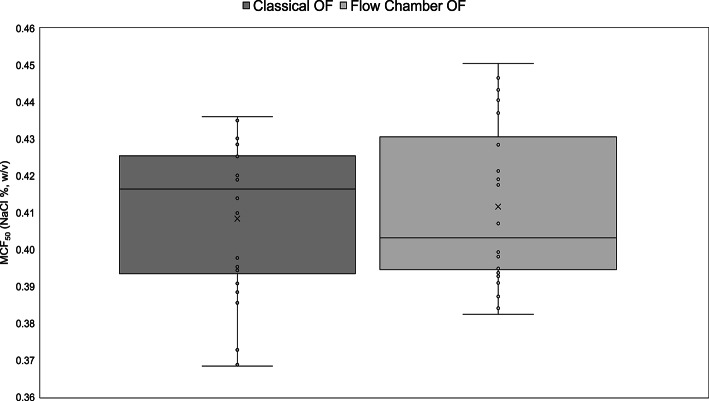
Box plots of MCF_50_ values obtained using the classical and flow chamber OF methods. The boxes show the interquartile range (IQR), the horizontal line indicates the median, and the “x” marks the mean. Individual data points (n = 24 per method) are represented by circles. No statistically significant difference was observed between the two methods (linear mixed-effects model with method as the fixed effect and donor as random intercept, *p* = 0.63).

As further illustrated in Fig. 4, the MCF_50_ values from the flow chamber method (BioExP OF) were highly consistent with classical OF test results. Both methods yielded identical mean values (0.41 ± 0. 02 (SD) % NaCl). These findings indicate that the flow chamber approach can reliably capture osmotic fragility parameters. The non-significant difference (p = 0.63) between the methods further supports the comparability of the two approaches.

To further evaluate whether the classical and the flow chamber OF measurement methods deliver consistent, mutually confirming results, a Bland–Altman analysis was performed^60^. Since the distribution of paired differences deviated significantly from normality (Q-Q plot and Shapiro–Wilk test, p = 0.001), a non-parametric approach was used^61,62^. In this context, the bias was calculated as the median of the paired differences, and the limits of agreement (LoA) were defined by the 2.5th and 97.5th percentiles, representing the range within which 95% of the differences between methods are expected to fall.

**Fig. 5:**
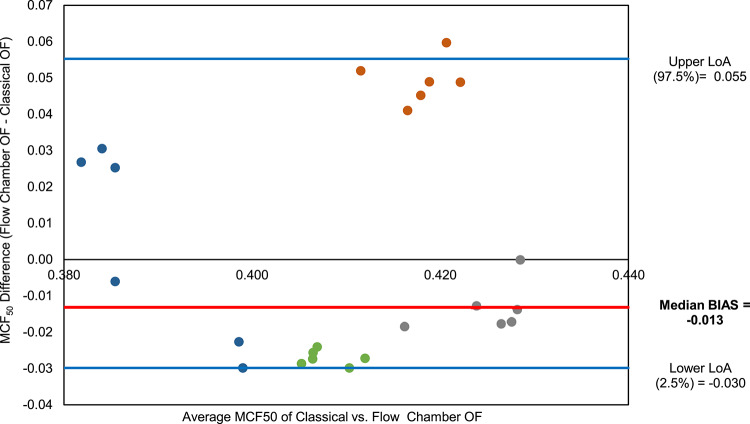
Non-parametric Bland–Altman plot comparing MCF₅₀ values obtained from the flow chamber and classical osmotic fragility methods. Points represent individual paired measurements (4 donors × 6 occasions) and are color-coded by donor. The y-axis represents the difference in MCF₅₀ values (Flow Chamber—Classical), while the x-axis shows the average of the two methods. The solid red line indicates the median bias (− 0.013), and the blue lines represent the 2.5th and 97.5th percentiles of the differences (− 0.055 and 0.030, respectively), serving as non-parametric limits of agreement.

The resulting Bland–Altman plot (Fig. 5) shows a median bias of –0.013, indicating that the flow chamber method, on average, yields slightly lower MCF₅₀ values compared to the classical approach. The 95% confidence interval (CI) for the median bias ranged from -0.027 to 0.025, indicating that the magnitude of systematic bias between the two methods is small. The non-parametric upper and lower LoA were 0.055 and –0.030, respectively. While one data point lay near the outer bounds, the majority were tightly clustered around the bias line, suggesting satisfactory agreement between methods.

Overall,  these results indicate that the flow chamber method performs comparably to the classical OF assay, demonstrating strong concordance with only minimal sample-level variation.

### Methods comparison based on LPS incubation and AQP inhibition experiments

**Fig. 6:**
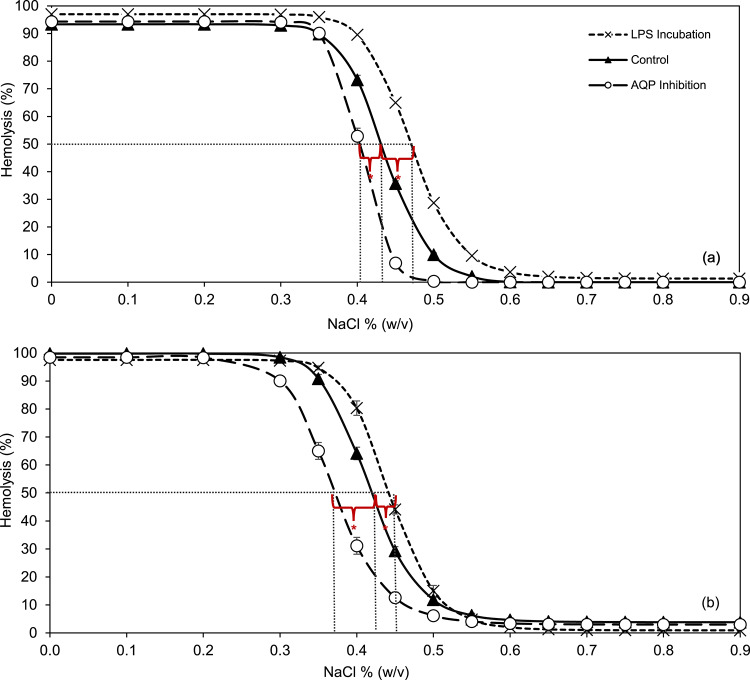
Fragility curves from a healthy donor showing control, LPS-incubated, and AQP-inhibited RBCs measured using the classical **(a)** and flow chamber **(b)** methods. Statistical significance was assessed using one-way ANOVA. AQP inhibition resulted in a significantly lower MCF₅₀ compared to the control group (*p < 0.001), while LPS incubation led to a significantly higher MCF₅₀ than control (* *p* < 0.001).

The effects of AQP inhibition and LPS incubation on OF were evaluated using both methods for comparison. Results are illustrated in the osmotic fragility curves (Fig. 6). Hemolysis curves were fitted using a four-parameter logistic function, and MCF₅₀ values were derived from the inflection point of each curve.

For the control group, the MCF₅₀ was 0.43 ± 0.004% NaCl and 0.42 ± 0.01% NaCl using the classical and the flow-chamber approach, respectively. Upon AQP inhibition with 40 µM HgCl₂, the MCF₅₀ shifted leftward, indicating reduced fragility, with values of 0.40 ± 0.01% NaCl (classical) and 0.37 ± 0.01% NaCl (BioExP). These reductions were highly significant compared to control (*p* < 0.001) for both methods. In contrast, LPS incubation resulted in increased fragility, as evidenced by a rightward shift of the curve, with MCF₅₀ values rising to 0.47 ± 0.004% NaCl (classical) and 0.44 ± 0.01% NaCl (flow chamber), both showing highly significant differences from control values (*p* < 0.001) (Fig. 6).

**Fig. 7:**
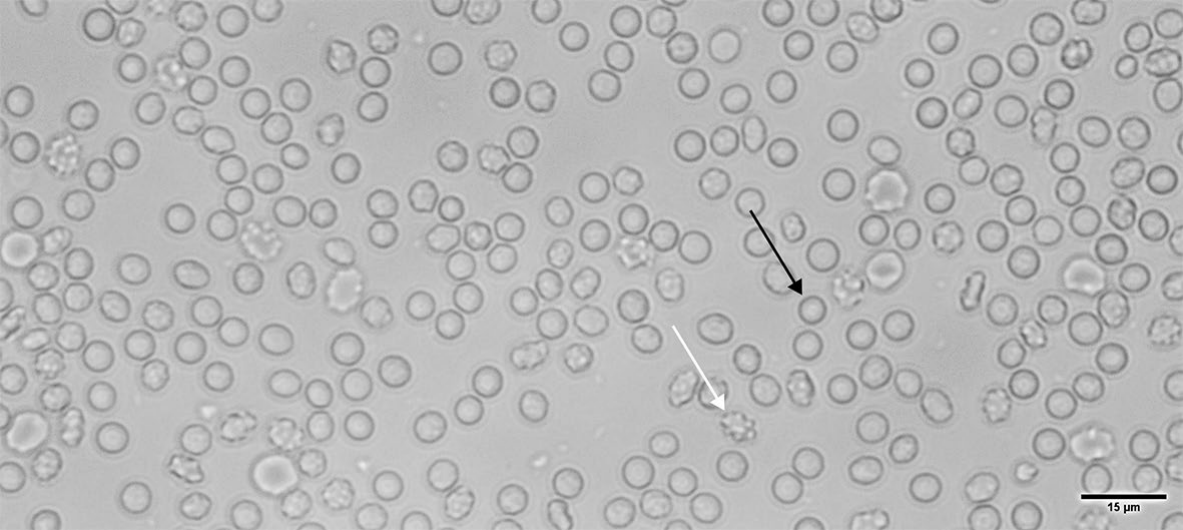
Representative image of the RBC monolayer at the start of the experiment in the flow chamber, incubated in isotonic solution (0.9% NaCl, w/v).Black arrow indicates a spherocyte, while the white arrow marks an echinocyte.

In Fig. 7, the morphological effects of LPS incubation on the RBCs are denoted with black and white arrows, showing spherocytic and echinocytic changes respectively. Notably, both the classical and flow-chamber methods consistently captured this biologically meaningful shift, reinforcing the sensitivity of the flow chamber system in detecting physiological changes in osmotic fragility.

**Fig. 8:**
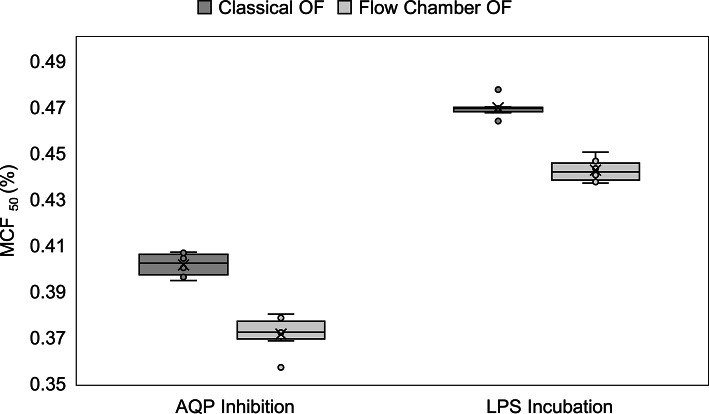
Box plot comparison of MCF_50_ values obtained using the classical and flow chamber OF methods for OF modulation via AQP inhibition and LPS incubation. The boxes show the interquartile range (IQR), the horizontal line indicates the median, and the “x” marks the mean. Individual data points (n = 6 repeated measures per method) are represented by circles.

Figure 8, on the other hand, presents box plots comparing MCF₅₀ values for AQP-inhibited and LPS-incubated RBCs, which shows that the flow chamber data are consistently lower than the classical OF data.

**Fig. 9:**
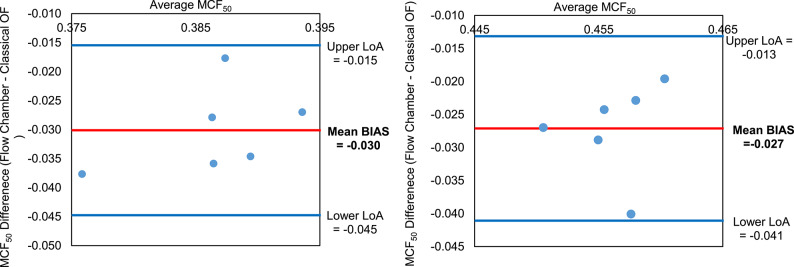
Parametric Bland–Altman plot comparing MCF₅₀ values obtained from the flow chamber and classical osmotic fragility methods for AQP inhibited and LPS incubated RBCs (**a**) and (**b**), respectively (single subject, n = 6 repeated, paired measurements). The y-axis represents the difference in MCF₅₀ values (Flow Chamber-Classical OF), while the x-axis shows the average of the two methods. The solid red line indicates the median bias (− 0.030 for AQP inhibited, − 0.027 for LPS incubated RBCs), and the blue lines represent the upper and lower LoA. The majority of points fall within this range, indicating an encouraging agreement between the two methods.

To further quantify this bias and assess agreement, separate Bland–Altman analyses were conducted for the AQP and LPS datasets (Fig. 9). Both showed a similar mean bias of approximately − 0.03% NaCl, consistent with the lower MCF₅₀ values observed in flow chamber measurements (Fig. 8). The upper and lower LoA were also comparable between conditions and methods, reinforcing the consistency and reliability of the flow chamber approach in detecting biologically relevant changes in osmotic fragility.

## Discussion

The osmotic fragility test is a well-established method in both research and clinical settings for assessing RBC membrane integrity and studying RBC water dynamics including AQP function^63,64^. Although simple, the classical OF assay is labor-intensive and time-consuming. Here, we present an innovative application of a custom-built flow chamber system coupled with AI-driven cell detection and automated OF calculation (BioExP). This platform enables rapid, high-throughput assessment of RBC osmotic fragility under tightly controlled experimental conditions, including temperature, shear flow and chemical exposure while simultaneously offering real-time imaging and documentation, that classical spectrophotometric methods cannot provide.

To adapt the classical OF testing to the flow chamber setup, several microscope slide coatings for optimal RBC adhesion were tested (data not shown). Poly-L-lysine coated slides were selected due to their ease of use, reliable performance and cost-effectiveness. Essentially, any other coatings could be used depending on the environmental conditions or disease models to be tested, making it broadly adaptable across experimental settings.

The second critical step in adapting the classical OF method to the BioExP involved the determination of the “satiation time”. This term refers to the incubation duration required for hemolysis to reach a near-steady state, ensuring that most susceptible RBCs have successfully lysed. As the RBCs are immobilized on the microscope slide, their reduced surface area exposed to the surrounding hypotonic solution likely slows water influx, hence hemolysis, compared to the cells in suspension. This makes the precise determination of the incubation/satiation time essential to maintain physiological relevance and measurement accuracy. As expected from the literature, hemolysis of the RBCs began almost immediately upon the application of hypotonic solution with a peak hemolytic response of 25.95% reached at approximately 2 min^58,59^. Approximately 77% of the total RBC lysis observed over 15 min occurred within the first 3 min of incubation. Based on this, a 3-min incubation was selected as optimal incubation time, balancing assay sensibility and accuracy while minimizing over- or underestimation of MCF_50_. This standardized approach not only ensures physiological relevance but also enables reproducible kinetic studies (with or without modulators) and provides unique opportunities for high-resolution video- and image-based analyses of RBC fragility dynamics.

To assess the agreement between the flow chamber (BioExP) and the classical OF assays, blood samples from four healthy donors were analyzed under control conditions. In clinical diagnostics, red blood cells from healthy individuals typically begin hemolysis at approximately 0.50% NaCl and complete lysis by around 0.30% NaCl (w/v)^65,66^. In our study, both the classical and flow chamber methods yielded an average MCF₅₀ of 0.41 ± 0.02% NaCl (w/v), aligning well with the expected physiological range. Key descriptive statistics, including SD, SEM and CV % between methods were nearly identical (CV = 5.19 and 5.21%, respectively), highlighting comparable precision and reproducibility. The slightly wider IQR of the flow-chamber method may be attributed to the variability due to surface attachment. Most importantly, statistical testing revealed no significant difference between the methods (*p* = 0.63), indicating agreement in MCF_50_ estimation.

Bland–Altman analysis revealed a small systematic bias with a narrow 95% CI for the median bias (− 0.027 to 0.025) indicating a small and consistent offset between the two methods. The differences between both methods largely remained within the upper and lower LoA. When accounting for repeated measurements within donors using the variance components approach, the corrected 95% LoA were -0.08 and 0.06, which were slightly wider as within-donor repeatability and between-donor heterogeneity are incorporated. This widening reflects donor-specific offsets in mean differences rather than increased measurement noise, consistent with the low within-donor variability observed across repeated measurements (Supplementary Table S1)^67^. The single outlier outside the limits of agreement is most likely attributable to random experimental variation rather than a systematic donor- or sample-specific effect (Fig. 5). Together with the absence of an apparent trend in the distribution of differences, these results suggest that the BioExP could serve as a robust and reliable tool for osmotic fragility testing under standard (control) conditions.

To further contextualize donor-level clustering observed in the Bland–Altman analysis, within-donor repeatability was quantified using donor-specific coefficients of variation and maximum absolute drift across repeated measurements (Supplementary Table S1). Repeatability analysis across six repeated measurements per donor showed low intra-donor variability (CV = 0.59–1.72% for the BioExP system and 0.67–4.87% for the classical OF method) and small maximum absolute MCF₅₀ drift values, typically 0.01–0.02 units (maximum 0.05). The highest drift was observed for a single donor and was captured by both methods (0.02 and 0.05 MCF₅₀ for the flow chamber and classical OF methods, respectively), yet remained limited in magnitude and consistent with expected day-to-day biological or experimental variability rather than systematic measurement instability. Together, these donor-level repeatability metrics indicate stable repeated measurements over time and support the interpretation that the observed agreement between methods primarily reflects true within-donor consistency. As an additional analysis, Spearman correlation was calculated for paired MCF_50_ values obtained with both methods, yielding ρ = –0.05 (p = 0.82). This low correlation, however, is expected given the narrow physiological range of MCF_50_ values. Therefore, our primary evaluation relied on Bland–Altman analysis.

To modulate the osmotic response of the RBCs, two physiologically relevant interventions were employed: (1) AQP inhibition, (2) LPS incubation of RBCs. For AQP inhibition a well-established thiol-reactive inhibitor of AQP, HgCl_2_ was chosen^36,68^. Given the known cytotoxic effects of mercury compounds, a concentration of 40 µM was selected based on prior dose–response experiments, where this dosage showed minimal evidence of PS externalization or echinocyte formation, however off-target effects can still reduce MCF_50_ ^57,69–71^. This allowed us to isolate the specific effect of inhibition on osmotic fragility without inducing morphological damage to keep AI-recognition viable.

Both the classical and flow chamber methods captured a significant leftward shift in the OF curve upon AQP inhibition (0.43 ± 0.004% NaCl and 0.42 ± 0.01% NaCl, respectively) in comparison to control values, consistent with previously published findings^30,72–74^. This confirms that under these conditions inhibition of aquaporin reliably reduced RBC fragility, a phenomenon captured robustly by both measurement systems.

In contrast, hemolysis of RBCs is a well-known complication in sepsis patients and is associated with increased mortality, though the underlying mechanisms remain multifactorial, ranging from Toll-like receptor 4 (TLR4)-mediated signaling to complement activation and possible direct membrane interactions^41,42,44^. Previous studies demonstrated that certain LPS serotypes with potent TLR4 agonist activity (stronger than the O55:B5 variant used in our study) can induce hemolysis by altering membrane composition and destabilizing lipid-protein interactions of RBCs^41–43^. Importantly, reports have also shown that LPS can increase fragility even in the absence of plasma or TLR4 signaling, suggesting a role for direct physiochemical effects on the RBC membrane^44^. Our findings align with this view: despite using a less potent serotype (O55:B5) and conducting experiments in plasma-free conditions, we observed a clear increase in RBC fragility. Notably, morphological alterations were already visible in the RBC monolayer at the start of the flow chamber measurements. As shown in Fig. 7, black arrow, the majority of RBCs exhibited a spherocytic morphology, accompanied by echinocyte transformations (white arrow), indicating membrane changes induced by LPS exposure. This points to non-immunological mechanisms, such as oxidative stress or membrane disruption, contributing to the observed effects. These morphological alterations were paralleled by a rightward shift in the OF curves, indicating that RBCs are more susceptible to lysis even at higher NaCl concentrations (0.47 ± 0.004% NaCl (classical) and 0.44 ± 0.01% NaCl (flow chamber/BioExP). The shifts induced by both AQP inhibition and LPS incubation were highly significant (p < 0.001), demonstrating the system’s sensitivity to both pharmacological and pathological conditions with direct relevance to clinical hematology.

The comparability of the two methods under modulation was further supported using Bland–Altman analysis. Both interventions showed similar negative bias between the flow chamber and classical methods (-0.030 for AQP inhibition; -0.027 for LPS incubation), indicating that the flow chamber consistently delivered lower MCF_50_ values (Figs. 8 and 9). This trend was also observed under control conditions from the same donor (bias: -0.013), suggesting the presence of a donor-specific measurement offset. Importantly, this directional consistency of the bias across all conditions strengthens the credibility of the flow chamber method in detecting relative changes in osmotic fragility. While absolute values may differ slightly between methods, the flow chamber/BioExP device reliably tracks physiological trends. This robustness against donor-specific variability (e.g., AQP expression, membrane integrity, hydration state, RBCage) underscores its potential for clinical application, where inter-individual differences are unavoidable.

All in all, the goal herein was to reduce the time, manual workload, and sample volume associated with the classical OF test. The classical OF requires extensive manual pipetting with high precision, spectrophotometric scanning and manual data processing. Depending on user experience and equipment, a single assay takes longer than 3 h from preparation to final logistic curve analysis. In contrast, BioExP system reduces the total assay time to 45–60 min per measurement, allowes kinetic testing and uses automated cell quantification, hemolysis calculation and logistic curve fitting. Furthermore, the flow chamber’s internal volume (approx. 230 mm^3^, with flow chamber height of 0.3 mm, width 14 mm, and length 55 mm) requires only minimal volumes of hypotonic solutions. These features collectively increase the number of samples that can be processed in a working day. Moreover, the AI-based single-cell detection rather than hemoglobin-dependent optical density measurements, further avoids signal saturation, tube-to-tube variability and volume sensitivity in spectrophotometric measurements. Also unlike ektacytometers, like LORRCA, this platform allows the user to test arbitrary numbers and concentrations of hypotonic solutions, without the constraints of predefined solution sets. The monolayer also enables direct manipulation of immobilized cells, with drugs, chemicals, temperature changes, shear stress etc. allowing real-time imaging of the same cells, something that is harder in bulk hemolysis or shear-based ektacytometry approaches. Although not yet methodically integrated into OF analysis, after modification of the current software, it will be in principle possible to analyze subpopulations of RBCs with different osmotic resistance and to determine the frequency distribution of the osmotic resistance of an RBC sample. This is currently the subject of further methodological developments.

Together, these findings demonstrate the proof-of-concept feasibility of using the BioExP platform for image-based osmotic fragility assessment. Considering the flow chamber system’s advantages such as reduced sample volume (just a finger prick), real-time imaging, higher throughput, and automation makes it suitable for both research and clinical use. Moreover, its ability to detect the effects of AQP inhibition and LPS exposure demonstrates translational sensitivity. Importantly, the BioExP system is not limited to these conditions, and any concentrations of pharmaceuticals, chemicals and modulators can be tested. Thus, the BioExP has the potential to serve as a diagnostic tool for studying hemolytic disorders (e.g. thalassemia, hereditary spherocytosis), transfusion medicine (RBC storage effects), sepsis-associated hemolysis, or therapeutic interventions as well as a versatile tool for mechanistic and temperature studies of RBC physiology. Future work could include parallel comparison with the widely used LORRCA Osmoscan to further contextualize the BioExP system. Although the classical and flow chamber methods showed encouraging agreement, extending the assessment across a broader donor cohort and operating conditions would support refinement and the calibration. Further, while the LPS and AQP modulation experiments illustrate the platform’s sensitivity, their restriction to samples from a single donor represents a limitation of the present study; extension to additional donors will enable broader translational interpretation. While the workflow is fully automated and therefore expected to exhibit low user-dependent variability, a brief inter-operator reproducibility assessment may offer additional insight to complement the automated workflow.

## Conclusion

This study presents an AI-integrated flow chamber platform (BioExP) for assessing red blood cell osmotic fragility, offering a modern, high-throughput, fast, kinetic and automated alternative to the classical spectrophotometric method. By integrating the monolayer technique with real-time imaging, flow control, and AI-based RBC detection, the system enables reproducible, low-volume MCF₅₀ determination, while offering flexibility in test conditions and the capability to assess a wide range of osmotic fragility-modulating factors. Comparison with the classical osmotic fragility assay across multiple healthy donors demonstrated strong agreement, supported by statistical comparisons including Bland–Altman analyses. Most importantly, the system was sensitive to biologically relevant modulators of RBC stability. AQP inhibition via HgCl₂ led to a significant decrease in RBC fragility, while LPS exposure, even in the absence of plasma, caused marked increases. These changes were consistently captured by both the classical and flow chamber methods, supporting the platform’s physiological accuracy for studying membrane dynamics under various conditions. Overall, the BioExP flow chamber system represents a promising, robust and versatile tool for osmotic fragility testing in both research and potential clinical contexts, combining precision, scalability, and biological relevance. This makes the BioExP device a good future candidate to become an integrative instrument in hematology laboratories, blood banks and more.

## Material and methods

1. Flow chamber system

The flow chamber consists of two parallel titanium plates fabricated using 3D printing. The upper plate features a circular window to enable real-time visualization under a microscope (VisiScope BL254T1, VWR, USA), equipped with a high-resolution camera mounted on the eyepiece (BRESSER MikroCam SP 5.0 microscope camera, Germany). The flow chamber has three inlet ports connected to a peristaltic pump system (ISMATEC, Switzerland) and a single outlet for fluid discharge. Degassing ports are included to minimize air bubble formation during operation. A Pt1000 temperature sensor is embedded within the chamber to monitor internal temperature, and the chamber is connected to an external temperature control unit for precise thermal regulation (LAUDA LOOP L100, Germany). The entire system is interfaced with PC and operated through LabManager (HiTec Zang, Germany). Experimental procedures and the AI data analysis are managed via proprietary software, BioExP Application, developed by HiTec Zang GmbH (Herzogenrath, Germany) and NeckarIT GmbH (Mössingen, Germany). Prepared solutions are stored in temperature-controlled fluid reservoir (TECAN, Germany) compatible with standard 50 mL laboratory tubes. Within the BioExP Application software, various standard operating procedures (SOPs) can be selected depending on the application. For OF testing SOP3 is used. The microscope slide within the chamber can be observed in real-time using the “Camera Live View” feature integrated into the software.

The AI was initially trained using a supervised learning approach. Images of RBC monolayers were captured and uploaded into the Label Studio platform, where individual RBCs were manually annotated using bounding boxes. For each experimental condition, a minimum of 10,000 RBCs were labelled to ensure sufficient confidence and training accuracy. These annotated datasets were then used by NeckarIT to train the AI model.

The trained AI was subsequently integrated into the BioExP Application software to enable automated quantification of RBCs in each image. By comparing the number of cells remaining after exposure to hypotonic solutions with the baseline monolayer, the system calculates the percentage of hemolysis for each NaCl concentration.

2. Blood collection

For the OF modulation experiments involving HgCl_2_ and LPS, blood was obtained from the first author via finger prick. For comparative measurements using both the classical and the flow chamber OF methods, blood was collected from four donors following standard laboratory procedures. Capillary blood was drawn via finger prick and collected into Na-heparin-coated microcapillaries (BRAND, Germany). The samples were centrifuged (Heraeus Biofuge Primo, Thermo Fisher Scientific, USA) three times at 1,000 × g for 10 min each to isolate RBCs. Hematocrit was measured using a microhematocrit centrifuge (Haematokrit 200, Hettich, Germany). All experimental procedures were reviewed and approved by the Ethics Committee of the Medical Faculty at RWTH Aachen University. The committee raised no ethical concerns and approved the study through a simplified review process, without requiring a formal vote. Blood samples were voluntarily donated with informed consent, in accordance with institutional guidelines and the Declaration of Helsinki.

3. Osmotic fragility modulation with AQP inhibition via HgCl_2_

To inhibit AQP activity, the well-established AQP inhibitor HgCl_2_ was chosen ^32,68,75^. A HgCl_2_ stock solution was prepared in phosphate buffered saline (PBS, pH 7.4). Isolated RBCs were resuspended at 20% hematocrit in PBS containing a final concentration of 40 µM and incubated at room temperature for 15 min. The selected concentration was based on our previous experiments using the classical OF method, where it significantly reduced RBC fragility without causing cytotoxic effects. Cell viability and morphology under these conditions were validated by assessing phosphatidylserine exposure via Annexin V staining, as well as quantifying echinocyte formation through microscopic imaging ^57^. These controls ensured that the observed reduction in fragility was due to functional AQP inhibition rather than morphological alterations. Following incubation, the samples were centrifuged to remove residual HgCl₂ and subsequently diluted with isotonic buffer to the target hematocrit level for use in the osmotic fragility experiments.

4. Osmotic fragility modulation with LPS

LPS (E.coli O55:B5, Sigma Aldrich, Merck, Germany) was used to induce increased OF in RBCs. Initial tests with lower LPS concentrations (300 mg/ml and 500 mg/ml) and shorter incubation times (2 and 4 h) produced minimal changes in MCF50 compared to control (data not shown). The following approach yielded the most pronounced change in fragility. Capillary blood was washed with PBS to remove plasma components, and the isolated RBCs were incubated at 10% hematocrit in PBS (290 mOsm, pH 7.4) containing 1000 mg/ml LPS for 24 h at 37 °C in a thermomixer (Thermomixer Compact, Eppendorf, Germany). Following incubation, residual LPS was removed by centrifugation, and the hematocrit was adjusted to the desired value for use in osmotic fragility experiments.

5. Classical osmotic fragility measurement

The classical OF method was employed to determine MCF_50_, defined as the NaCl concentration where 50% hemolysis occurs. The procedure was based on the standard protocol established by Parpart et al.^1^. Fifteen aqueous NaCl solutions (pH 7.4) were prepared at the following concentrations: 0.90%, 0.80%, 0.75%, 0.70%, 0.65%, 0.60%, 0.55%. 0.5%, 0.45%, 0.4%, 0.35%, 0.30%, 0.2%, 0.1% and 0% (w/v). The osmolarities of these solutions were measured via a cryoscopic osmometer (OSMOMAT 030, Gonotec, Germany). The hematocrit of the blood samples were adjusted to 36% and equal volumes of blood were added to each NaCl concentration in a 1:100 dilution ratio. The samples were periodically mixed and incubated at room temperature for 30 min. Following this, they were centrifuged to separate the supernatant. The absorbance of each supernatant was measured at 540 nm using a UV–VIS spectrophotometer (V550, JASCO, Japan). The percentage of hemolysis was calculated using the formula; % Hemolysis = (O.D. of the test sample—O.D. of 0.90% sample) / (O.D. of 0% sample—O.D. of 0.90% sample) × 100. A logistic curve was fitted to the resulting hemolysis data using MatLab (MathWorks, USA) to calculate MCF_50_ value.

6. Flow chamber osmotic fragility measurement

This protocol was developed in our laboratory following extensive optimization using the BioExP flow chamber system and measurements were done at room temperature. The first step of the measurement is to build the RBC monolayer. To be able to adapt osmotic fragility assay we determined the time required to achieve near-complete hemolysis at a NaCl concentration of 0.4%, a concentration that typically corresponds to ~ 50% hemolysis in classical OF assays. This time point was defined as the “satiation time” and was subsequently used for all experimental conditions. After ensuring the connections of the BioExP system, the gaskets were properly positioned inside the flow chamber, and a clean microscope slide (Thermo Fisher Scientific, USA) was inserted into the chamber. The BioExP Application software was launched, and the peristaltic pump was activated at 48 rpm using isotonic buffer (PBS) to clean the chamber and prime the tubing. Following this initial priming, the slide was replaced with a clean poly-L-lysine-coated microscope slide (Thermo Fisher Scientific, USA). A 30 µL blood sample, adjusted to 1% hematocrit, was pipetted onto the slide, and the chamber was sealed. An initial PBS flow was (48 rpm) was applied to create a uniform RBC monolayer. The quality of the monolayer was assessed under the microscope (VisiScope BL254T1, VWR, USA) equipped with a high-resolution camera (BRESSER MikroCam SP 5.0). The RBCs were allowed to sediment and attach undisturbed for 15 min to promote adequate adhesion. After this, the monolayer was briefly washed (48 rpm) with PBS to remove non-adherent RBCs. An initial image of the adhered RBC monolayer was captured using the software to serve as a reference (0.9% NaCl). Before switching to each new hypoosmolar NaCl solution, the tubing and connectors were flushed to remove dead volume and prevent mixing of solutions. The next hypotonic solution (e.g., 0.80%) was then introduced at 48 rpm for 20 s. Flow was stopped, and the monolayer was incubated statically with the solution for 3 min. A subsequent image was captured to assess the extent of hemolysis at that specific concentration. To reverse the cumulative osmotic stress, an isotonic PBS wash was performed after each hypotonic treatment. PBS was flushed through the system at 48 rpm for 20 s, followed by a 1.5-min incubation with the isotonic solution. This cycle was repeated for each NaCl concentration used in the classical OF until complete hemolysis of the monolayer was achieved. All images were saved and labelled in the software according to their corresponding NaCl concentration. Upon completion of the experiment, the microscope slide was removed, and the system was thoroughly cleaned by flushing with PBS followed by ethanol before each new trial.

7. AI model training and validation

To train and validate the AI a dataset was curated and annotated. Ground truth annotations were created manually using bounding boxes for detection tasks and polygon masks for segmentation tasks. The dataset was characterized by high object density. Although consisting of fewer images (N = 36), it contained over 14,000 annotated cells, reflecting the densely packed monolayers required for fragility testing. To ensure robust generalization, the dataset was divided into training (80%), validation (10%), and testing (10%) subsets using a greedy stratified sampling strategy. For object detection tasks (fragility classification), the YOLOv7 architecture was employed due to its balance of speed and accuracy. To improve robustness against occlusion and varying cell densities, aggressive augmentations were applied including Mosaic and MixUp (probability 0.15), as well as Paste-in (0.15) to simulate overlapping cells. Geometric transformations included translation (+ -20%) and scaling (gain 0.9). Model performance was evaluated on the held-out test set using standard COCO metrics: Precision (0.926), Recall (0.937), and mean Average Precision at IoU thresholds of 0.5 (0.946) and 0.5:0.95 (0.561).

8. Statistical analysis

Descriptive statistics were performed for all experimental data. The dataset comprised four healthy donors, each sampled on six separate occasions, resulting in a total of n = 24 paired measurements. For Bland–Altman analysis samples were paired for classical and the BioExP system. Inferential comparisons between methods were conducted using a linear mixed-effects model with method as a fixed effect and donor as a random intercept. Agreement between the classical and flow chamber-based OF methods was assessed using Bland-Altmann method (normality of differences were tested with Shapiro–Wilk test), to evaluate consistency, bias, the limits of agreement (LoA). Finally, to assess the effects of HgCl_2_ and LPS on OF within a single donor one-way ANOVA followed by Tukey–Kramer post hoc tests was conducted for each method (classical and flow chamber) separately. Prior to ANOVA, normality was assessed using Shapiro–Wilk test, and the variance homogeneity was evaluated using the Levene’s test. All statistical analyses were conducted using Microsoft Excel and GraphPad Prism.

## Supplementary Information

Below is the link to the electronic supplementary material.

Supplementary Material 1

## Data Availability

Data are available from the corresponding author upon request.
