# Supplementary material for: A novel AI-coupled flow chamber method quantifying erythrocyte osmotic fragility
Source: Sci Rep. 2026 Feb 17;16:7175. doi: 10.1038/s41598-026-38322-z (PMC12920795; doi:10.1038/s41598-026-38322-z)
Supplement: Supplementary file 1 — Supplementary Material 1 [file 41598_2026_38322_MOESM1_ESM.pdf]

A novel AI-coupled Flow Chamber Method Quantifying Erythrocyte Osmotic Fragility and Rapid Effects of AQP inhibition and Lipopolysaccharide Exposure

Ipek Seda Firat[1], Özgür Alacayir[1], Till Creutz[1,2], Gerhard Michael Artmann[1], Samar Damiati[3], Aysegül Temiz Artmann[1]

[1] Center of Competence for Bioengineering, University of Applied Sciences Aachen, Medical and Biological Laboratory, Jülich, Germany (firat@fh-aachen.de)

[2] HiTec Zang GmbH, TPH Technology Park Herzogenrath / Aachen, Germany

[3] Department of Chemistry, College of Sciences, University of Sharjah, Sharjah, United Arab Emirates (sdamiati@sharjah.ac.ae)

SUPPLEMENTARY MATERIAL

Supplementary Table S1 *Repeatability metrics of MCF_{50} values across repeated measurements for each donor. Standard deviation (SD) and coefficient of variation (CV %) quantify within-donor variability across repeated measurements, with CV % calculated as SD divided by the mean. Maximum absolute drift represents the largest absolute difference observed between repeated measurements within the same donor. (n = 6 repeated measurements per donor; repeated-measures design).*

Donor	Method	Mean MCF_{50}	SD	CV%	Max Drift (absolute)
1	BioExP	0.42	0.01	1.51	0.02
1	Classical	0.43	0.00	0.92	0.01
2	BioExP	0.44	0.00	1.09	0.01
2	Classical	0.39	0.00	1.03	0.01
3	BioExP	0.39	0.00	0.59	0.01
3	Classical	0.42	0.00	0.67	0.01
4	BioExP	0.39	0.01	1.72	0.02
4	Classical	0.39	0.02	4.87	0.05
